# Late Results of Early End-to-End Repair in Sciatic Nerve Injuries

**DOI:** 10.7759/cureus.47101

**Published:** 2023-10-16

**Authors:** Azmi Tufan

**Affiliations:** 1 Department of Neurosurgery, Kadıköy Florence Nightingale Hospital, Istanbul, TUR

**Keywords:** tibial nerve injury, peroneal nerve injury, end-to and anastomosis, anastomosis, sciatic nerve injury, sciatic nerve

## Abstract

Objective: Mechanisms of sciatic nerve injury include gunshot injury, sharps injury, injection injury, contusion, femoral fracture injury, and iatrogenic injury due to fracture surgery. Regardless of the type of injury, patients undergoing sciatic nerve repair have poor motor and sensory outcomes. In this study, we compared the late outcomes of a group of patients in whom the author performed an early end-to-end anastomosis for sciatic nerve sharps injury and another group of patients with a similar injury who were not operated on but left to natural history.

Methods: The sciatic nerve, comprising two primary divisions with distinct muscle innervations, was subject to separate examinations. Group 1 (n=10, study group) underwent tibial division anastomosis, while Group 2 (n=12, control group) received no surgical intervention involving the tibial division. Similarly, Group 3 (n=11, study group) underwent peroneal division anastomosis, while Group 4 (n=14, control group) encompassed subgroups that did not undergo peroneal division surgery.

Results: In Group 1, the rate of gain in plantar flexion muscle strength was significantly higher (p < 0.05) compared to Group 2. Furthermore, the sensory examination gain level ratio within the tibial domain was significantly greater (p < 0.05) in Group 1 than in Group 2. Additionally, Group 1 exhibited a significantly higher rate (p < 0.05) of detection of regeneration and reinnervation findings in electromyography (EMG) compared to Group 2.

Conclusion: When evaluating the long-term outcomes following early end-to-end anastomoses of the sciatic nerve, it becomes evident that while significant improvements are observed when compared to individuals without anastomosis, the positive impact of surgical interventions on motor and sensory gains in daily life remains limited. Nevertheless, we contend that early surgical intervention holds potential advantages in terms of patient management.

## Introduction

The causes of sciatic nerve injuries vary based on cultural factors and a country's developmental status. Mechanisms include gunshot wounds, injuries from sharp objects, injection-related traumas, contusions, femur fracture-associated injuries, and iatrogenic complications from fracture surgeries. Despite the injury type, clinical outcomes consistently pose challenges, marked by diminished motor and sensory function [1]. Each type of sciatic nerve injury leads to distinct clinical trajectories based on the patient's presentation. A common practice in peripheral nerve surgery involves a multi-phase approach, starting with a three-week waiting period post-injury to assess nerve damage. Clinicians then observe for signs of regeneration and reinnervation over three months. However, this conventional tracking has limitations, as rapid degeneration and retraction occur in both the distal and proximal regions of the injury site post-peripheral nerve injury [2]. Late-stage surgical intervention becomes unfeasible due to nerve retraction, preventing primary repair. End-to-end nerve repair with grafts, either autogenous or heterogeneous, raises issues of graft placement, leading to suboptimal clinical outcomes and technical challenges. Surgeons often delay intervention for sciatic nerve injuries, especially those with partial damage, to acquire electrophysiological evidence before proceeding. The infrequent occurrence of surgeries related to sciatic nerve injuries, often performed under emergency circumstances, contributes to a lack of surgical experience in managing these cases promptly [3].

Injuries caused by sharp cutting tools present a distinct clinical scenario for sciatic nerve injuries. Early surgical intervention in this subset holds promising potential for favorable outcomes, particularly in preserving neural continuity. This contrasts sharply with challenging proximal nerve injuries, where achieving successful surgical results is hindered. The theoretical prospects of successful intervention are markedly higher when executed in the early phases of injury, prior to substantial nerve retraction, emphasizing the critical importance of timely interventions for optimal clinical outcomes [4].

Extensive studies on surgical interventions for sciatic nerve injuries often lack a focused examination, encompassing various injury mechanisms and surgical modalities collectively. Moreover, these studies frequently lack standardized outcome measures, introducing potential variability and ambiguity. A notable gap in the literature is the absence of studies undertaking a comparative analysis between surgical treatment and natural healing outcomes within similar patient cohorts. This scarcity highlights a significant gap, hindering a nuanced understanding of the efficacy and limitations of surgical approaches in specific clinical contexts. Systematic reviews generally express dissatisfaction with surgical outcomes for sciatic nerve injuries [1]. However, notable disparities exist in the scientific literature, with some individual studies reporting elevated success rates. This highlights the need for a comprehensive examination of factors contributing to divergent findings and the identification of variables influencing surgical management outcomes for this condition [3-6].

The researcher, an accomplished surgeon with expertise in peripheral nerve surgery [7-9], conducted a comparative analysis involving two patient groups. One group underwent early end-to-end anastomosis surgery for sciatic nerve lacerations induced by cutting tools, while the other was managed conservatively. Despite modest sample sizes, the study's uniqueness lies in the meticulous examination of a specific injury type and the outcomes associated with a particular surgical technique. Focusing on this subset of patients contributes valuable insights into the clinical implications of early end-to-end anastomosis for sciatic nerve injuries induced by cutting tools, enhancing understanding and highlighting its clinical relevance.

## Materials and methods

This investigation involved a comprehensive evaluation of patients treated at the Brain and Nerve Surgery Clinic of Bağcılar Training and Research Hospital between 2010 and 2021. This study titled 'Late-term results of early end-to-end anastomosis of the sciatic nerve' was carried out with the permission of Medipol University Clinical Research Ethics Committee (Date: 31.08.2023, Decision No: 704). The study encompassed a total of 12 cases who underwent surgical interventions conducted by the author in response to sciatic nerve lacerations during the specified period. Additionally, 14 cases, followed without surgical intervention for various reasons, were sourced from the electrophysiology laboratory records of the same hospital over an identical timeframe. Notably, the sciatic nerve comprises two primary divisions, each innervating distinct muscle groups, necessitating separate assessments of the tibial and peroneal divisions.

The patient cohort was categorized into four distinct groups, each delineated by specific criteria. Group 1 (n=10, study group) comprised individuals who underwent tibial division anastomosis, while Group 2 (n=12, control group) encompassed patients who did not receive tibial division surgery. Similarly, Group 3 (n=11, study group) represented individuals subjected to peroneal division anastomosis, while Group 4 (n=14, control group) involved patients within the peroneal division, segregated into four subgroups, including those who did not undergo surgical intervention. Comparative analyses were performed between Group 1 and Group 2, as well as between Group 3 and the aggregated Group 4. Demographic data of all patients were meticulously recorded, providing a comprehensive profile of the study population. Muscle strength assessments adhered to the established British Medical Research Council (BMRC) system (Table 1).

**Table 1:** BMRC scale for muscle strength (0-5) BMRC: British Medical Research Council

Grade 0	No visible contraction
Grade 1	Visible contraction without movement of the limb (not existent for hip flexion)
Grade 2	Movement of the limb but not against gravity
Grade 3	Movement against gravity over (almost) the full range
Grade 4	Movement against gravity and resistance
Grade 5	Normal

Sensory evaluations were conducted employing the Semmes-Weinstein monofilament test (Table 2). Electrophysiological studies (EMG) yielded findings categorized based on the presence or absence of reinnervation and regeneration indicators, adding an objective dimension to the assessments.

**Table 2:** Semmes-Weinstein monofilament test s2PD: Static sence of two-point discrimination (mm); m2PD; motor sence of two-point discrimination (mm).

Grade	Recovery of sensibility	s2PD, m2PD
S0	No recovery of sensibility in the autonomous zone of the nerve	
S1	Recovery of deep cutaneous deep sensibility within the autonomous zone of the nerve	
S1+	Recovery of superficial pain sensibility	
S2	Recovery of superficial pain sensibility and some touch sensibility	
S2+	As in S2, but with overresponse	
S3	Recovery of superficial pain sensibility and some touch	with > 15 > 7 sensibility
S3+	As S3, localization of the stimulus is good and there is imperfect recovery	7-15 4-7
S4	Complete recovery	2-6 2-3

Inclusion criteria

Eligibility criteria for enrollment in the study group encompassed individuals who presented with a complete or near-complete incision injury affecting at least one division of the sciatic nerve. Further stipulations included prompt hospital admission within the initial 24 hours following the injury, discernible muscle groups demonstrating graded strength of either 1/5 or 0/5, and the performance of primary end-to-end anastomosis within the initial 48 hours following the traumatic event. Prospective participants in the study group were also required to commit to a comprehensive clinical and electrophysiological follow-up regimen spanning a minimum duration of two years.

Conversely, eligibility criteria for inclusion in the control group consisted of individuals who sustained incision injuries resulting in complete or near-complete nerve damage within at least one division, as evidenced by early electrophysiological studies. Similar to the study group, individuals in the control group were also subject to a stringent clinical and electrophysiological follow-up protocol spanning a minimum period of two years. These criteria were meticulously established to ensure the rigorous and equitable evaluation of both study cohorts.

Exclusion criteria

The study rigorously established uniform exclusion criteria for both the study and control groups to uphold the homogeneity of the study population. These criteria encompassed the exclusion of individuals with injury patterns divergent from those resulting from incisions, ensuring the exclusive enrollment of participants with sciatic nerve injuries attributable to incisional causes. Furthermore, individuals displaying evidence of partial nerve injuries, as indicated by muscle strength assessments of 2/5 or higher, were systematically excluded from the study cohorts. This criterion aimed to concentrate the investigation on individuals with complete or near-complete nerve damage, preserving a consistent level of injury severity within the study population. Lastly, individuals unable to commit to a comprehensive clinical and electrophysiological follow-up regimen spanning a minimum duration of two years were excluded from both study groups. This temporal prerequisite was instituted to facilitate a comprehensive, long-term assessment of outcomes, reinforcing the robustness and reliability of the study's findings.

Anatomy and surgical technique

The sciatic nerve, originating from the confluence of five spinal nerve roots spanning from L4 to S3, stands as the most substantial and lengthy peripheral nerve within the human body. It serves a multifaceted role, encompassing both sensory and motor functions, contributing significantly to the functionality of the lower limb. Morphologically, the sciatic nerve exhibits a broad and flat configuration within the hip region, progressively assuming a more cylindrical shape as it descends towards the lower extremity, eventually spanning an approximate length of 2 centimeters.

Within the context of this study, the sciatic nerve is discretely divided into three distinct segments based on the location of injury. The upper part pertains to injuries originating from the sciatic notch and extending to the region traversing the gluteus maximus muscle. Middle-part injuries involve damage to the deep-seated component of the nerve, commencing from the inferior border of the gluteus maximus and extending beneath the biceps femoris muscle. In the case of lower part injuries, the sciatic nerve's divisions are isolated, with the tibial division injury site defined up to its entrance into the popliteal fossa, while the peroneal division injury site is delineated until it enters the bony groove.

Regardless of the precise location of the incision-induced injury, a surgical approach that adheres to the anatomical trace, prioritizing the preservation of skin vascularity, is consistently employed. Following the meticulous dissection of muscular layers to expose the nerve, the knee is flexed and stabilized to alleviate tension. The use of automatic retractors facilitates the surgical procedure, conducted under the guidance of a microscope. Initial assessments involve the verification of the continuity of both nerve divisions, with probing conducted using a nerve probe instrument. In cases of concomitant vascular injuries, vascular surgeons perform the necessary vascular repairs.

For primary nerve anastomosis, the nerve endings intended for reconnection are meticulously approximated, ensuring a precise end-to-end alignment of nerve fibers. Thick sutures, typically 2.0 silk suture material (26 mm round; Dogsan, Turkey), are employed to epineurally approach either the tibial or peroneal branch at a 120-degree angle from the posterior aspect. This choice of suture material is made to mitigate any potential dissection of the epineural tissue by nylon sutures. Recognizable nerve fibers are subsequently anastomosed in an end-to-end fashion through the placement of two stitches each, employing 9/0 nylon suture material (5.0 mm round; Dogsan, Turkey). It is worth noting that the primary method employed for anastomosis is through epidural sutures. The remaining 240-degree section of the nerve and the gaps between the thick sutures in the remaining 120-degree section are meticulously sutured using 4.0 silk material (13 mm round; Dogsan, Turkey). Following the closure of anatomical layers, the knee is immobilized at an approximate 90-degree angle without extension, effectively preventing undue tension on the sutures, a crucial factor in preventing anastomotic failure.

Statistical analysis

Mean, standard deviation, median, lowest, highest, frequency and ratio values were used in the descriptive statistics of the data. The distribution of variables was measured with the Kolmogorov-Simirnov test. Paired sample t-test and Wilcoxon test were used in the analysis of dependent quantitative data.

## Results

The study encompassed a total of 26 patients, with a gender distribution of 15.4% male and 84.6% female. Among the 26 patients who underwent surgical interventions, 10 received tibial division anastomoses, and 11 underwent peroneal division anastomoses, resulting in 12 patients receiving surgical treatment. The mean duration of follow-up was 49.3±20.4 months. Conversely, among the 14 patients who did not undergo surgery, 11 presented with tibial division injuries, and all exhibited peroneal division damage. Notably, the distribution of nerve injury sites within the cohort was 13.6%, 50.0%, and 36.4% from proximal to distal, respectively.

The incidence of late neuropathic pain was reported at 8.3% within the anastomosis groups, while the non-surgical groups exhibited a notably higher rate of 28.5%. Within the anastomosis groups, Group 1 displayed a significantly higher (p < 0.05) rate of improvement in plantar flexion muscle strength compared to Group 2. Moreover, the rate of gain in sensory examination levels within the tibial division was notably higher (p < 0.05) in Group 1 compared to Group 2. Additionally, the detection rate of regeneration and reinnervation findings in electromyography (EMG) examinations significantly favored (p < 0.05) Group 1 over Group 2 (Table 3).

**Table 3:** Comparison between Group 1 and Group 2 X² Chi-square test.

Tibial division	Grade	Group 1			Group 2		p	
		n	%		n	%		
The level of muscle strength achieved, plantar flexor	0	1	10.0%		9	75.0%	0.002	^X²^
	I	3	30.0%		3	25.0%		
	II	5	50.0%		0	0.0%		
	III	1	10.0%		0	0.0%		
The level of sensory evaluation achieved, tibial trace	0	1	10.0%		7	58.3%	0.019	^X²^
	I	7	70.0%		5	41.7%		
	II	2	20.0%		0	0.0%		
Evidence of regeneration and reinnervation in EMG	(-)	1	10.0%		6	50.0%	0.045	^X²^
	(+)	9	90.0%		6	50.0%		

Conversely, the comparison between Group 3 and Group 4 within the non-surgical cohorts revealed no significant differences (p > 0.05) in plantar flexion muscle strength gain. However, Group 3 exhibited a significantly greater (p < 0.05) rate of sensory examination improvement compared to Group 4. Furthermore, EMG examinations yielded significantly higher (p < 0.05) rates of detecting regeneration and reinnervation in Group III when contrasted with Group 4 (Table 4).

**Table 4:** Comparison between Group 3 and Group 4 X² Chi-square test.

Peroneal division	Grade	Group 3			Group 4		p	
		n	%		n	%		
The level of muscle strength achieved, dorsal flexor	0	4	36.4%		10	71.4%	0.080	^X²^
	I	4	36.4%		4	28.6%		
	II	2	18.2%		0	0.0%		
	III	1	9.1%		0	0.0%		
The level of sensory evaluation achieved, peroneal trace	0	3	27.3%		11	78.6%	0.01	^X²^
	I	5	45.5%		3	21.4%		
	II	3	27.3%		0	0.0%		
Evidence of regeneration and reinnervation in EMG	(-)	4	36.4%		11	78.6%	0.032	^X²^
	(+)	7	63.6%		3	21.4%		

## Discussion

It is imperative to acknowledge that the prevailing body of scientific literature addressing sciatic nerve injuries has often amalgamated various injury etiologies, treatment timings, and methodologies, presenting a comprehensive but potentially heterogeneous perspective. In contrast, this study specifically delves into a select subset of cases characterized by incision-induced injuries, wherein primary end-to-end repair was employed as the chosen treatment modality. By focusing exclusively on this distinct injury category, our investigation provides a tailored and in-depth analysis, thus diverging from the broader, more generalized assessments encountered in the existing literature. It is noteworthy that while both our study and control groups consist of relatively modest sample sizes, they harbor a distinctiveness attributed to their exclusive relevance to end-to-end repair of sciatic nerve injuries resulting from incisions. This specificity endows our study with a singular vantage point, offering unparalleled insights into the outcomes associated with this particular treatment approach. Furthermore, the scarcity of comparable studies within the existing literature facilitated our ability to engage in meaningful comparisons with a limited number of analogous investigations. Although constrained by the paucity of directly analogous studies, this analytical approach has permitted us to extract valuable insights that enrich our understanding of the efficacy and outcomes of end-to-end repair in the context of sciatic nerve injuries induced by incisions.

Within our study, notable improvements were observed in late-term outcomes pertaining to muscle strength, sensory examination, and electrophysiological findings among individuals who underwent tibial division anastomosis (Group 1) when contrasted with the control group. In the peroneal anastomosis group (Group 3), a statistically significant enhancement was evident in sensory examination results and electrophysiological findings compared to the control group, although muscle strength improvement, while notable, did not achieve statistical significance. In light of the longest-standing series documented in the literature, our study findings bear a noteworthy resemblance [4]. For instance, in a study conducted by Murovic JA [4], favorable outcomes for tibial division anastomoses were reported at 79% at the hip level and 90% at the thigh level. Similarly, success rates for peroneal division anastomoses in Murovic's study stood at 55% at the hip level and 61% at the thigh and knee levels. This aligns with our observations, suggesting that peroneal division repairs may exhibit a relatively more limited degree of recovery in comparison to tibial division repairs. Further corroborating this trend, Roganovic Z reported recovery rates of 11%, 31%, and 57% for proximal-to-distal sciatic repairs resulting from gunshot wounds [10]. In the context of our study results and observations, it becomes evident that peripheral nerve recovery rates tend to diminish as the distance from the injury site to the target organ increases [11]. In instances of complete sciatic nerve transection, despite the implementation of end-to-end repair techniques, there exists a rapid degeneration process in both the proximal and distal nerve segments. In essence, the severed nerve assumes an inactive state and primarily serves as a scaffold, secreting chemical mediators conducive to the regeneration of new nerve fibers. This phenomenon elucidates the rationale behind the decline in recovery rates as the distance from the target organ augments, highlighting the intricate nature of peripheral nerve regeneration processes.

Consistently across various injury mechanisms, including those analyzed in our study, the tibial division demonstrates a notably heightened capacity for recovery when juxtaposed with the peroneal division. This divergence may be attributed, in part, to inherent anatomical disparities. Specifically, the peroneal division's lateral and superficial course, coupled with its fixation at two distinct points, the hip and fibular groove, render it more susceptible to injury. Furthermore, its relatively limited surrounding supportive tissue may contribute to the heightened vulnerability observed in this division. However, these anatomical considerations, while influential, are insufficient to fully elucidate the intricacies surrounding the challenges encountered in peroneal division recovery. It is conceivable that a healing tibial division, when successfully repaired, can crudely innervate proximate muscles such as the gastrocnemius and soleus, given their relative proximity. In contrast, for a peroneal division that has undergone repair proximal to the knee, achieving beneficial reinnervation and regeneration poses a more formidable challenge. This stems from the necessity for the peroneal division to traverse a physically restrictive tunnel, such as the fibular groove, impeding its progress. Furthermore, the peroneal division must extend its innervation to a multitude of more distal muscles, intensifying the complexity of the recovery process [12].

Within our study, it was observed that the plantar and dorsal flexor muscle strengths, in association with both tibial and peroneal divisions, failed to surpass a grade 3 rating according to established clinical grading systems. Notably, the most prevalent outcome within the tibial division subgroup was an augmentation of motor strength by two levels, accounting for 50% of cases. Furthermore, an enhancement of sensory examination findings by one level was documented in 58.3% of cases. Comparatively, the peroneal division exhibited slightly weaker outcomes, with fewer cases attaining the aforementioned improvements in motor and sensory domains. These findings underscore the challenges associated with achieving higher levels of motor and sensory recovery in the context of tibial and peroneal divisions, shedding light on the nuanced nature of peripheral nerve recovery within this specific cohort. It is noteworthy that some previous studies [5,13] have reported instances of notable motor strength improvement, denoted by a ratio of 4 or above, particularly in the context of primary tibial anastomosis, where a 5/6 ratio was observed. Similarly, primary peroneal anastomosis exhibited improvements, albeit with a 3/6 ratio.

Of particular interest is the observation that in cases where end-to-end repair was performed on nerves initially presenting with a motor strength level of 0/5, the attainment of motor strength levels reaching 4 and 5 represents a remarkable and transformative development. This underscores the potential for substantial motor recovery in scenarios characterized by severe initial deficits, highlighting the dynamic and adaptive nature of peripheral nerve regeneration processes. A fundamental aspiration in peripheral nerve repair pertains to the establishment of uninterrupted continuity within the endoneural tubes. This imperative endeavor is further underscored by the intricate and dynamic nature of nerve regeneration, where new nerve fibers extend towards diverse muscle groups, ultimately contributing to the orchestration of coordinated movements that approximate normal function. Despite the advanced microsurgical methodologies at our disposal, it is essential to approach these outcomes with a sense of guarded optimism. The formidable challenges stem from the inherent limitations associated with nerve regeneration. Specifically, even with meticulous microsurgical expertise, regenerated axons lack the capacity to selectively navigate to their original endoneural tubes. Furthermore, the regenerated nerve fibers are confronted with the arduous task of seeking out targets that had not been reached during their initial innervation [14]. Furthermore, it is imperative to acknowledge the constraints surrounding the re-proliferation of denervated muscle fibers. These limitations add another layer of complexity to the multifaceted challenge of achieving functional recovery following peripheral nerve injuries [15]. An intriguing facet of this challenge relates to the assessment of sensory and motor outcomes using standardized scales. Specifically, the inherent subjectivity in evaluating the resistance capacity of muscles engaged in gravity-defying actions poses a substantial methodological quandary. This subjectivity introduces variability among assessors, potentially leading to divergent interpretations of similar muscle strength. To enhance the scientific discourse in this domain, it becomes imperative to emphasize the value of comprehensive and meticulous patient reports. Specifically, documenting cases wherein patients achieve normal muscle strength subsequent to primary end-to-end repair is of paramount significance.

Within the study groups, a notable occurrence of reinnervation findings was evident, with rates of 90% and 63.6% observed in electromyographic (EMG) studies for the tibial and peroneal divisions, respectively. Conversely, in the control groups, comparatively lower reinnervation rates were noted, standing at 50% for the tibial division and 21.4% for the peroneal division. While it is established that unrepaired sciatic nerves often culminate in neuroma formation, the emergence of relatively high reinnervation rates warrants further consideration. This phenomenon may be attributable to misdirected regeneration, a form of aberrant reinnervation that arises from neighboring healthy nerves rather than stemming from the regeneration of the initially damaged nerve. It is important to note that these findings align harmoniously with the extant literature, substantiating the notion that misdirected regeneration, as a manifestation of aberrant reinnervation, plays a discernible role in the peripheral nerve regeneration process [16].

During the late period of our study, the utilization of splints was observed in four out of 12 cases within the study group, and in seven out of 14 cases within the control group. It is noteworthy that despite a relatively higher incidence of splint usage among individuals in the non-surgical group, it is plausible to infer that the decision to employ splints may be influenced by individual preferences, appearing somewhat independent of the motor strength exhibited.

The presence of late-onset neuropathic pain was documented in one out of 12 cases within the study group and four out of 14 cases within the control group. These findings illuminate a compelling trend, suggesting that cases involving nerve transection, particularly those managed without surgical intervention, are more susceptible to culminating in the development of painful neuromas in the post-injury period.

Upon a comprehensive examination of the study results, it becomes evident that the outcomes following sciatic nerve end-to-end anastomosis, while significantly superior in comparison to the control groups, may still be characterized as suboptimal with regard to the attainment of meaningful muscle strength and sensory enhancements. One plausible explanation for the observed limitations in surgical success rates may pertain to the intricacies inherent to the procedure. Despite our endeavors to execute epiperineural repair without inducing tension, it is noteworthy that we were only able to perform two or three fascicular repairs in select cases. This highlights the inherent challenges associated with achieving tension-free fascicular anastomosis, even in cases where considerable surgical expertise is applied. In light of these considerations, it is imperative to underscore the emerging concept of supermicrosurgery as a potential avenue for enhancing outcomes in peripheral nerve repair. Supermicrosurgery represents a microneurovascular anastomosis technique tailored to vessels measuring between 0.3 and 0.8 mm, as well as single nerve fascicles. Its application scope is rapidly expanding across diverse domains, encompassing nerve reconstruction, reimplantation procedures, amputated fingertip reconstruction, microsurgical flap salvage, and free tissue transfer. The integration of supermicrosurgical techniques may hold promise in addressing the challenges encountered in peripheral nerve repair, offering new avenues for improved functional outcomes [17]. The acquisition of proficiency in the supermicrosurgery technique represents a prospective avenue for augmenting the outcomes achieved in the realm of peripheral nerve surgery. The adoption of this specialized technique holds promise in addressing the complexities and challenges inherent to such surgical interventions. It is worth contemplating the potential influence of the postoperative splinting regimen on our surgical outcomes. Notably, our employment of a flexion splint for a duration of only three weeks following surgery may have contributed to suboptimal results. Noteworthy findings from prior research indicate that a more extended postoperative period under the influence of flexion splinting, spanning six weeks with the knee maintained at a 90-degree flexion angle, yielded substantial motor and sensory improvements. These findings, surpassing the outcomes observed in our study, underscore the importance of an extended splinting regimen as a factor that merits further investigation and consideration in the context of peripheral nerve surgery [6].

Upon conducting a comparative analysis of our study outcomes against a limited selection of studies available in the literature, it becomes apparent that the extent of improvement observed in our results, particularly concerning final muscle strength and sensory examination levels, exhibits a relatively more restrained profile. Despite our diligent examination, we have not identified any objective factors that could comprehensively account for this outcome, except for potential contributing factors such as our utilization of fascicular repair techniques and the relatively brief duration of postoperative splint usage. It is noteworthy, however, that our cumulative experience prompts us to critically evaluate cases where end-to-end repair yields full strength gain, as occasionally reported in the literature [5]. Such exceptional outcomes, which appear to contradict the fundamental principles governing peripheral nerve healing, merit scrutiny. This critique arises from the recognition that surgeons, especially those new to the domain of peripheral nerve repair, may inadvertently misinterpret limited improvements in their cases as instances of failure when compared to the exceptionally positive outcomes documented in the literature. It is imperative to acknowledge that the realm of sciatic nerve end-to-end anastomosis surgery remains fraught with challenges, particularly in cases involving proximal injuries. Despite the advancement of surgical techniques, achieving optimal results in such scenarios remains an ongoing quest. Nevertheless, the findings from our study underscore the potential advantages associated with early end-to-end anastomosis in incisional injuries affecting the sciatic nerve, offering a valuable perspective for clinicians and researchers in the field.

Limitations of the study

The most important limitation of this study, which reports the results of a relatively rarely applied surgical technique, is that the number of cases is insufficient to reach strong statistical results.

## Conclusions

Upon a comprehensive evaluation of the late-stage outcomes stemming from early end-to-end anastomoses of the sciatic nerve, a discernible pattern emerges. While evidence substantiates a significant degree of improvement in comparison to cases devoid of anastomosis, the translation of these motor and sensory enhancements into tangible improvements in the daily lives of patients appears somewhat limited. Nonetheless, it remains our contention that the timely application of surgery, particularly in the early stages, contributes to the more effective management of patients grappling with sciatic nerve injuries. We advocate for a nuanced perspective on the exceptionally favorable outcomes occasionally reported in select end-to-end anastomosis studies, urging a tempered assessment due to the intrinsic complexities governing peripheral nerve regeneration and muscle reinnervation mechanisms. It is essential to recognize that, notwithstanding the advancements in microsurgical techniques, the contemporary landscape of peripheral nerve surgery, particularly within the domain of proximal nerve injuries, continues to be characterized by suboptimal outcomes. These enduring challenges underscore the need for ongoing research and innovation, aiming to ameliorate the functional recovery experienced by patients affected by sciatic nerve injuries.
